# Human Umbilical Cord Mesenchymal Stem Cells Attenuate Severe Burn-Induced Multiple Organ Injury via Potentiating IGF-1 and BCL-2/BAX Pathway

**DOI:** 10.1155/2022/5474289

**Published:** 2022-12-23

**Authors:** Hongyu Wang, Te Ba, Qiong Wang, Longlong Yang, Chenyi Li, Xingxia Hao, Yue Yin, Lingying Liu

**Affiliations:** ^1^Department of Burn Surgery, The Third Affiliated Hospital of Inner Mongolia Medical University (Inner Mongolia Bao Gang Hospital), Baotou, 014010 Inner Mongolia, China; ^2^Department of Burn and Plastic Surgery, The Hohhot First Hospital, Hohhot, 010000 Inner Mongolia, China; ^3^Senior Department of Burns and Plastic Surgery, The Fourth Medical Center of PLA General Hospital, 100037 Beijing, China; ^4^Department of Nutrition, The Fourth Medical Center of PLA General Hospital, Beijing 100037, China; ^5^The Inner Mongolia Medical University, Hohhot, 010110 Inner Mongolia, China

## Abstract

**Background:**

Early multiple organ injuries induced by severe burn predict a high mortality. Mesenchymal stem cells (MSCs) are able to repair and reconstruct the injured tissues and organs induced by trauma and diseases. However, potential protective effect and mechanism of MSCs on multiorgan injury induced by severe burn at early stage remain to be not clarified. Therefore, this study was to explore the effect and mechanism of human umbilical cord-derived MSCs (hUCMSCs) against severe burn-induced early organ injuries in rats.

**Methods:**

Adult male Wistar rats were randomly divided into sham, burn, and burn+hUCMSCsgroups. GFP-labeled hUCMSCs or PBS was intravenous injected into respective groups. Migration and distribution patterns of GFP-labeled hUCMSCs were observed by inverted fluorescence microscope. The structures and cell apoptosis of the heart, kidney, and liver were measured by immunohistochemistry. Biochemical parameters in serum were assayed by standard Roche-Hitachi methodology. Western blotting was performed on these organs of rats in the three groups to explore the underlying mechanisms.

**Results:**

At 24 hours after hUCMSCs transplantation, we found that GFP-labeled hUCMSCs mainly localized in the blood vessel of the heart, kidney, and liver and a very few cells migrated into tissues of these organs. Compared with the sham group, structure damages and cell apoptosis of these organs were induced by severe burn, and systematic administrations of hUCMSCs significantly improved the damaged structures, cell apoptosis rates, and biochemical parameters of these organs. Furthermore, IGF-1 (insulin-like growth factor 1) level in burn+hUCMSCs group was significantly higher than that in the sham and burn groups. Meanwhile, severe burn induced BCL-2/BAX significantly decreased compared to the sham group, and it was markedly increased by hUCMSCs administration.

**Conclusion:**

The hUCMSCs transplantation can attenuate severe burn-induced early organ injuries and protect multiorgan functions by encouraging migration of hUCMSCs with blood circulation and increasing protective cytokine IGF-1 level and regulating BCL-2/BAX pathway of these vital organs. Furthermore, these data might provide the theoretical foundation for further clinical applications of hUCMSCs in burn areas.

## 1. Introduction

Multiorgan injury is a serious complication post severe burn and has a propensity to progress into multiple organ dysfunction syndrome (MODS), even multiple organ failure (MOF) [1]. The pathogenesis of multiorgan injury post severe burn is multifactorial. Firstly, oxidative stress directly and quickly causes structural damage and cell apoptosis of multiple organs post severe burn [2, 3]. During subsequently burn-induced shock phase, reperfusion injury further results in secondary damage of these organs [4]. Furthermore, endogenous proinflammatory cytokines and coagulation pathways post severe burn are hyperactive and out-of-balance also added seriously to organ damages [5–7]. These comprehensive factors contribute to multiorgan injury, dysfunction, even failure, collapse of the circulatory system, and eventually death [8, 9]. Although there are some treatments in the clinic, actively effective therapies for preventing organ damages are still lacking and urgently needed in early stage post severe burn.

Numerous studies have demonstrated that local or systemic administration of MSCs can reduce severe disease-associated or severe trauma-induced organ injuries and mortality rates in relevant animal models [10, 11]. This may be a consequence of ameliorating the pathological alterations and reducing the production of proinflammatory cytokines in animal models [12, 13]. Recent clinical trials have preliminarily demonstrated that hUCMSCs therapy for COVID-19, ARDS, and pulmonary fibrosis was very effective and safe [14, 15]. It is worth noting that MSCs-based therapies have been considered to be promising strategies to treat patients with severe trauma and its main complications through secretion of soluble paracrine protein factors and exosomes [16–18]. Therefore, the aim of the present study is to evaluate the potential protective effects of hUCMSCs on organ injuries in severely burned rats and relevant mechanisms.

## 2. Materials and Methods

### 2.1. Animal Care

All studies adhered to the procedures consistent with the International Guiding Principles for Biomedical Research Involving Animals issued by the Council for the International Organizations of Medical Sciences (CIOMS) and were approved by the Institutional Animal Care and Use Committee at the Fourth Medical Center Affiliated to PLA General Hospital. Six-week-old male Wistar rats (weighing 180–220 g) were anesthetized by intraperitoneal injection of 300 mg/kg Avertin (20 mg/mL) (2,2,2-tribromoethanol, Sigma, USA) [19–21].

### 2.2. Isolation, Phenotypic Identification, and Labeling of hUCMSCs

The study was approved by the ethical committee of the Fourth Medical Center Affiliated to PLA General Hospital. Umbilical cord tissues (15-20 cm) from full-term healthy fetus with phosphate-buffered saline (PBS) were cut into 1-3 mm tissue blocks after removal of the umbilical vessels and external membrane. The tissue blocks were placed in culture flasks at a distance of 0.5 cm, added with mesenchymal stem cell medium-serum free (MSCM-sf) (ScienCell Research Laboratories, San Diego, CA) at 37.0°C in a humidified atmosphere of 5% CO_2_. The medium was replaced slowly and slightly every 3 days, and it was made sure that the tissue block was fixed. When the cells around these tissue blocks reached 80% to 85% confluence, the tissue blocks were removed, and these cells were digested with trypsin-EDTA and transferred to T-75 culture bottles for propagation and culture. The morphology of hUCMSCs of primary passage (P0), P1, and P4 was observed using inverted phase contrast microscope (Leica Microsystems, Wetzlar, Germany).

The fourth-passage hUCMSCs were collected, washed twice in PBS, and digested with trypsin-EDTA. After the final wash in PBS, a single cell suspension (1.0 × 10^6^ cells per 100 *μ*L) was added to each tube. Tubes 1 and 2 consisted of negative controls, while fluorescent-labeled mouse antihuman antibodies (1 : 100), including FITC-CD105, FITC-CD90, FITC-CD44, PE-CD29, FITC-HLA-I, FITC-CD45, FITC-CD34, FITC-CD11b, FITC-CD31, FITC-vWF, PE-CD19, and PE-HLA-DR were added to the remaining tubes, respectively. These experimental tubes were incubated in the dark at 37°C for 30 minutes, centrifuged, washed twice with PBS, and detected using flow cytometer (FCM) (Becton Dickinson, Franklin Lakes, NJ). Then, hUCMSCs from passages 4–8 were used for all experiments.

Three days before transplantation, hUCMSCs were labeled with green fluorescent protein (GFP) using a lentiviral strategy [22]. The lentivirus vector system was constructed, packed, and purified by GeneChem (Shanghai, China). The positive rate of GFP-labeled hUCMSCs was assayed by flow cytometer (FCM).

### 2.3. Animal Model of Severe Burn and Treatments

The 30 adult male Wistar rats were randomly divided into 3 groups (*n* = 10): sham, burn, and burn-transplanted hUCMSCs. A model of 50% TBSA and full-thickness burn was prepared. After the rats were anesthetized by intraperitoneal injection of Avertin (300 mg/kg), the dorsal and abdominal hair was removed completely, first with clippers and then through the application of Veet depilatory cream. Both whole backside and abdomen were, respectively, placed in hot water (94°C) for 12 s and 6 s, which caused 50% TBSA with a full-thickness burn [23]. An immediate injection of balanced salt solution (40 mg/kg) was administered to prevent shock. The wound was then treated with 1% tincture of iodine and kept dry to prevent infection. The rats in the sham group were placed in water at 37°C for 12 s, and the other processes were the same as those applied to the burned rats. Wounds were left open and animals were sacrificed at defined time points post severe burn [21].

The rats in the burn-transplanted hUCMSCs group immediately received a tail vein injection of 5 × 10^6^/1 mL GFP-labeled hUCMSCs after severe burn. The rats in the other groups received a tail vein injection of 1 mL PBS at the same time.

### 2.4. Specimen Collection and Detection

The blood samples of the abdominal aorta were taken at 24 h post severe burn. They were transferred immediately to coagulator-containing tubes, and serums for analyzing biochemical parameters were collected by centrifugation. Meanwhile, samples of the vital organs such as the heart, kidney, and liver were also collected. These samples were carefully removed and rinsed in PBS. Then, every specimen was divided into three pieces: one piece was prepared into frozen section and observing locations of GFP-labeled hUCMSCs, another piece was stored in liquid nitrogen for future molecular detection, and the last remaining part piece was fixed in 4% paraformaldehyde for future immunohistochemical staining detection.

### 2.5. Migration and Distribution of GFP-Labeled hUCMSCs

GFP-labeled hUCMSCs were applied to the severely burned rats to detect the migration and distribution patterns of hUCMSCs in vivo. To evaluate whether hUCMSCs migrated into the injured organs or not, tissues of the heart, kidney, and liver were prepared into frozen section for tracking GFP-hUCMSCs using inverted fluorescence microscope.

### 2.6. Histological Staining

After fixation with 4% paraformaldehyde for 1 week at room temperature, the specimens were embedded in paraffin and sectioned in a plane perpendicular to the incision. Five-micrometer-thick sections were prepared, deparaffinized in dimethylbenzene, and rehydrated. Preparative sections were stained with H&E in accordance with the standard procedures. Other sections were stained with In Situ Cell Death Detection Kit (Roche Applied Science, Germany) following the directions of the manufacturer. The apoptotic nuclei were shown to be brown. The cell apoptosis rates in the heart, kidney, and liver tissues were counted in 5 randomly selected fields of the each slide by an experienced and independent cell scientist in a blinded manner.

### 2.7. Western Blotting

The proteins of the heart, kidney, and liver of rats in the three groups were immediately extracted using RIPA buffer supplemented with Halt protease inhibitor cocktail (Servicebio, Wuhan, China). Proteins were subjected to SDS-PAGE gel, and anti-BCL-2 and anti-BAX antibodies (1 : 1,000) and anti-IGF-1 and anti-*β*-actin antibodies (1 : 2,000) (R&D Systems, Minneapolis, MN) were used for protein expression assay.

### 2.8. Statistical Analysis

All data are expressed as the mean ± SD and were analyzed using SPSS 18.0 (SPSS Inc., Chicago, IL, USA). Statistical differences among the groups were assessed by one-way ANOVA, and post hoc multiple comparisons were performed using the Student-Newman-Keuls tests. The significance level was set at *P* < 0.05.

## 3. Results

### 3.1. Isolation, Identification, and Labeling of the hUCMSCs

**Figure 1:**
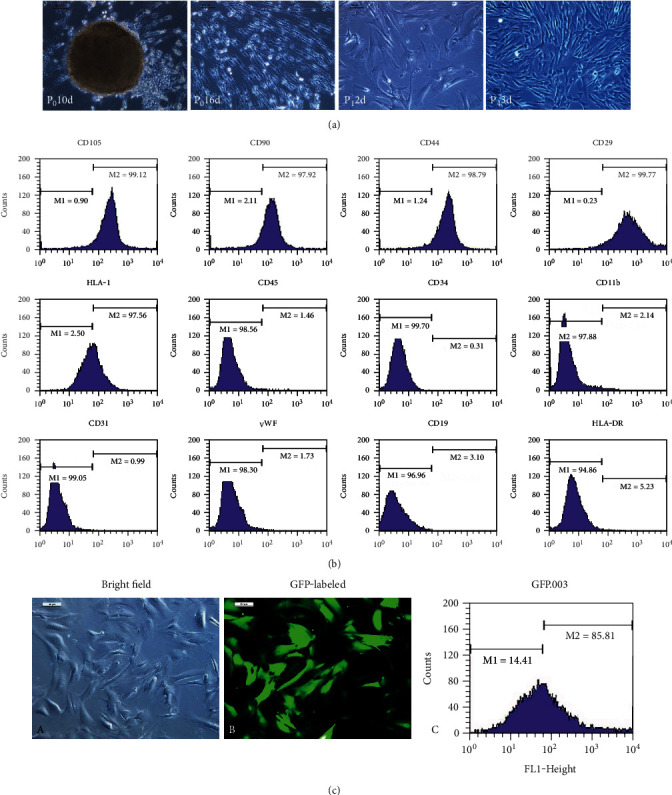
The hUCMSC isolation, culture, and identification. (a) The hUCMSCs were isolated and cultured by adherent method, as well as grew in vortex shape. (b) The hUCMSCs are positive expression of CD105, CD90, CD44, and CD29 and negative expression of CD45, CD34, CD11b, CD31, vWF, CD19, and HLA-DR using flow cytometry. (c) The hUCMSCs were labeled with green fluorescent protein (GFP) using a lentiviral strategy (GFP-hUCMSCs).

At day 8-10 of the tissue block attachment, long fusiform cells were observed in the gaps between the tissue blocks. The cells around the tissue blocks reached 90% to 95% confluence, about day 16 of culturing. Morphology of the isolated and cultured primary cells (P0) was similar to fibroblasts. These cells were primarily polygonous at P1 and gradually changing into a uniform spindle shape at 4th passage (Figure 1(a)).

Cellular immune phenotypes were determined using flow cytometer. The cells were positive for the cell surface markers CD105, CD90, CD44, CD29, and HLA-I but were negative for the cell surface markers CD45, CD34, CD11b, CD31, vWF, CD19, and HLA-DR (Figure 1(b)). These results were consistent with the immune-phenotypic markers of MSCs.

The hUCMSCs from passages 4–8 had a uniform spindle shape, and stable biological properties were in their logarithmic multiplication cycle. The hUCMSCs were labeled with GFP using a lentiviral vector strategy. Our data demonstrated that this strategy is highly effective in producing infectious viral particles expressing GFP (88.37 ± 4.53%) in grafted hUCMSCs (Figure 1(c)).

### 3.2. Migration and Distribution of GFP-Labeled hUCMSCs

**Figure 2:**
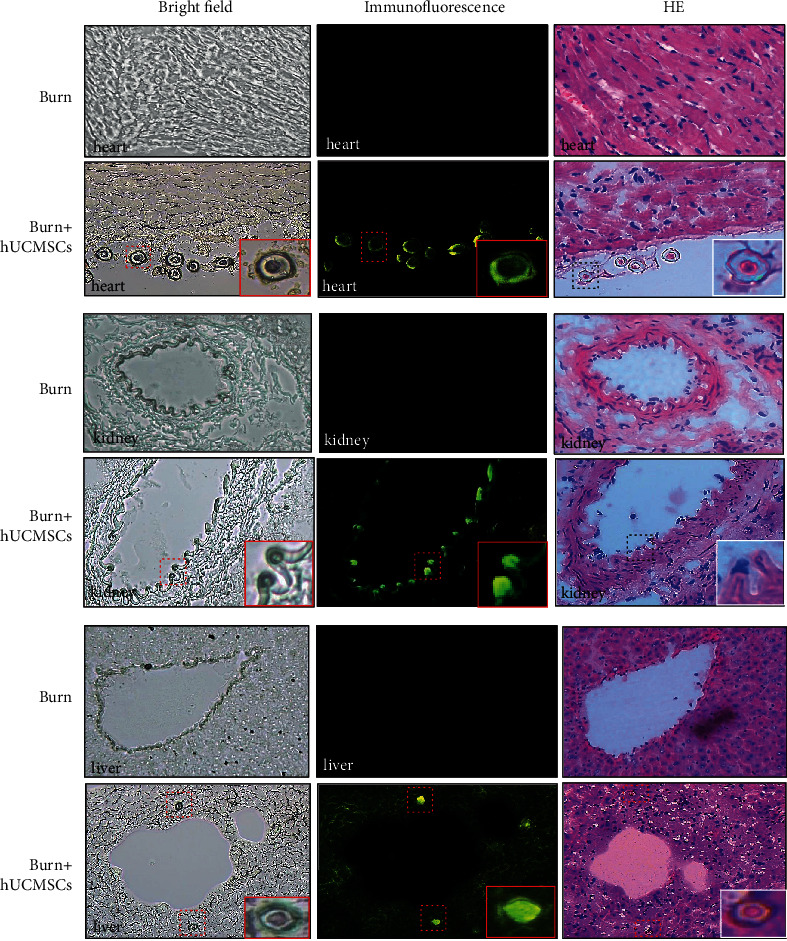
GFP-hUCMSCs were tracked in severely burned rats *in vivo*. The GFP-labeled hUCMSCs mainly migrated and distributed in blood vessels of the heart, kidney, and liver, and a very few cells migrated into tissues of these organs.

To assess whether GFP-labeled hUCMSCs migrated into the injured organs induced by severe burn or not, tissue specimens of the heart, kidney, and liver were prepared into frozen sections for observing migration and distribution pattern of GFP-labeled hUCMSCs using inverted fluorescence microscope. At 24 hours after cell transplantation, we observed that GFP-labeled hUCMSCs mainly concentrated in the blood vessels of the heart, kidney, and liver, and a very few cells migrated into the tissues of these organs. However, we have not found fluorescence signal of hUCMSCs in rats of the burn group (Figure 2).

### 3.3. The hUCMSCs Administration Alleviates Structure Damage of Vital Organs

**Figure 3:**
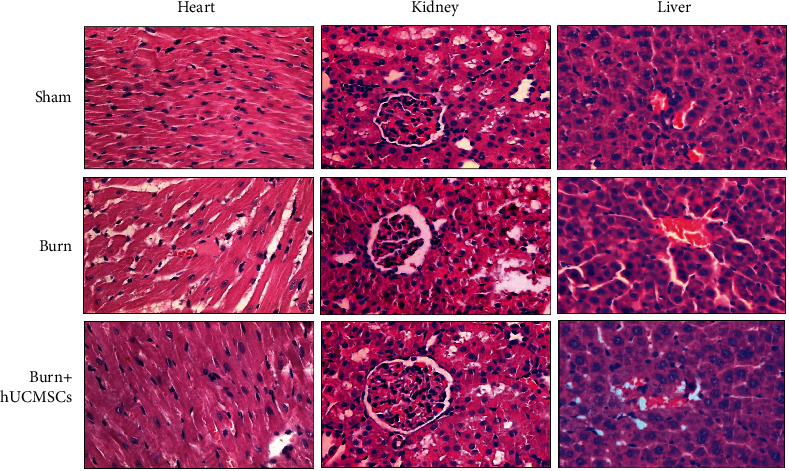
The hUCMSC administrations could significantly improve severe burn-induced structural damages of vital organs, such as the heart, kidney, and liver.

To investigate the effect of hUCMSCs administration on damaged structures of the heart, kidney, and liver at 24 h post severe burn, we evaluated structure changes of these organs using H&E staining. As shown in Figure 3, compared with the sham group, severe myolysis, vacuolar degeneration and sarcoplasm agglutination of the heart, dilated mesangial area of glomeruli and ischemia of capillary loops of the kidney, and hepatocellular swelling and vacuolar changes of the liver were induced by severe burn. However, hUCMSCs administration could significantly improve structural damages of these vital organs.

### 3.4. The hUCMSCs Administration Reduces Cell Apoptosis Rates of Vital Organs

**Figure 4:**
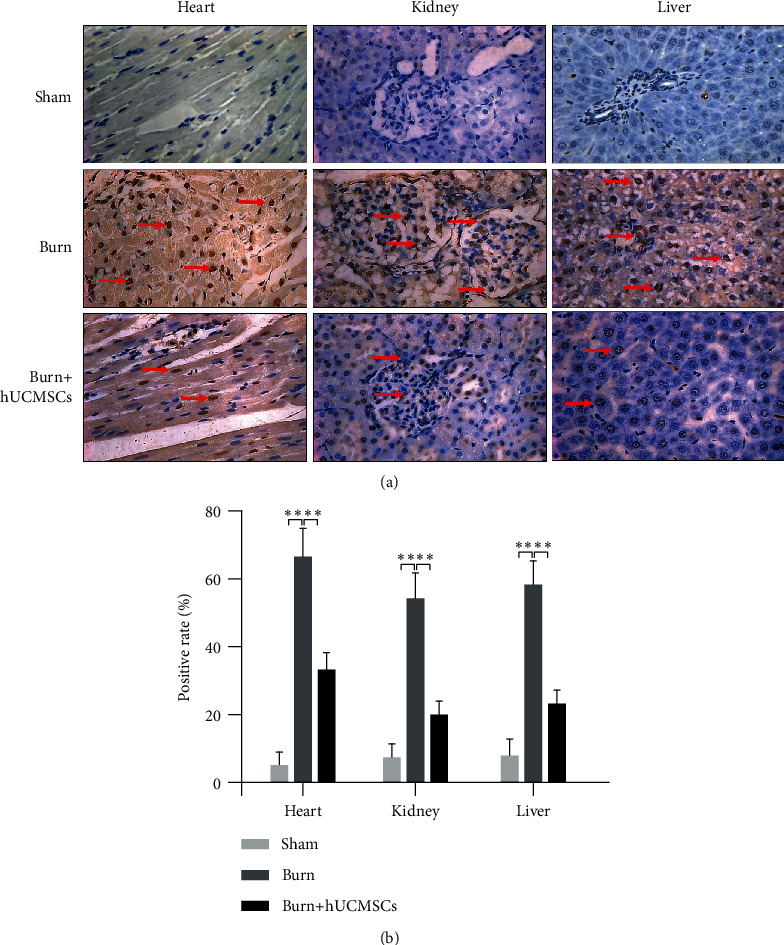
The hUCMSC administrations could significantly improve severe burn-induced cell apoptosis rates of the heart, kidney, and liver. (a) Cell apoptosis rates of vital organs in the burn group were significantly higher than those in the sham group, and they were significantly decreased in the burn+hUCMSC group than those in the burn group. (b) The result of the quantitative analysis is presented in the corresponding histogram.

To evaluate the effect of hUCMSCs administration on cell apoptosis rates in the heart, kidney, and liver at 24 h post severe burn, we assayed cell apoptosis rates of these vital organs in 3 groups using TUNEL staining. As shown in Figure 4(a), cell apoptosis rates of the heart, kidney, and liver in the burn group were markedly higher than that in the sham group, and they in the burn+hUCMSCs group were significantly lower than that in burn group. The result of the quantitative analysis is presented in the corresponding histogram (Figure 4(b)).

### 3.5. The hUCMSC Administration Improves Biochemical Parameters in Serum

The serum samples of the rats in the 3 groups at 24 h post severe burn were analyzed for the crucial and sensitive biochemistry parameters as summarized in Table 1. As high sensitive parameters of myocardial damage, troponin, CK, and CK-MB levels in burn group were explicitly increased than those in sham group, and they were significantly decreased by hUCMSCs administration. In addition, we also found that the levels of liver function parameters ALT, AST, and LDH and the levels of kidney function parameters urea and creatinine in the burn group were markedly higher than those in the sham group, and their levels in the burn+hUCMSCs group were significantly lower than those in the burn group.

### 3.6. The hUCMSCs Administration Increases Protective Cytokines

**Figure 5:**
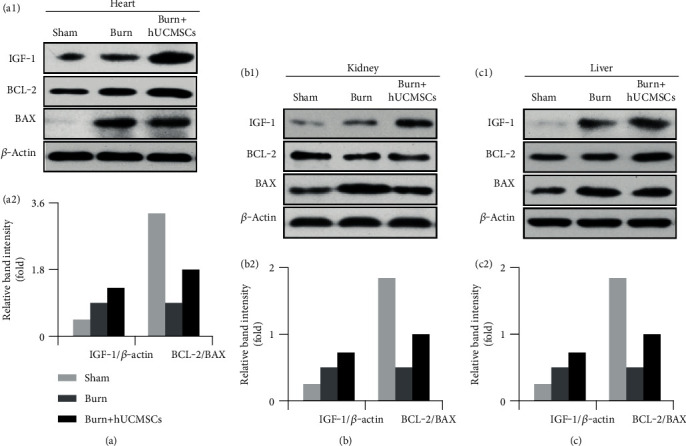
The hUCMSC administrations significantly increased protective cytokine IGF levels and BCL-2/BAX ratios. (a1–c1) IGF-1 levels of the heart, kidney, and liver in the burn+hUCMSC groups were significantly higher than those in the burn groups, and they in the burn group were higher than those in the sham group. BCL-2/BAX ratios of the heart, kidney, and liver in the burn groups were markedly lower than those in the sham groups, while they in the burn+hUCMSC groups were significantly higher than those in the burn groups. (a2–c2) The results of the quantitative analysis were presented in the corresponding histograms.

To elucidate the potential mechanism responsible for the effect of hUCMSCs on protection of these injured organs, we next tested the levels of the antiapoptosis cytokine IGF-1 and apoptosis-related proteins, such as BCL-2 and BAX in the heart, kidney, and liver in the three groups. As shown in Figure 5(a), IGF-1 levels of the burn+hUCMSCs groups in the heart, kidney, and liver tissues were significantly higher than those in the sham and burn groups. Furthermore, BCL-2/BAX of the burn groups in the heart, kidney, and liver tissues was markedly lower than those in the sham groups, while BCL-2/BAX in the burn+hUCMSCs groups was significantly higher than those in the burn and sham groups. The result of the quantitative analysis is presented in the corresponding histogram (Figure 5(b)).

## 4. Discussion

Burns are one of the most common injuries in times of peace and war. The World Health Organization estimates that over 265,000 deaths result annually from burns, with over 95 percent occurring in low- and middle-income countries [24]. In China, burns are the second leading injury after trauma caused by the traffic accident. And severe burn-induced tissues and organ injuries could lead to multiple organ dysfunction syndrome (MODS) and multiple organ injury multiple organ failure (MOF), even death [9, 25]. Burn severity generally directly correlates to the total body surface area (TBSA) and burned deep of the injured tissue [26]. Above 30% TBSA of deep burns in adult and 10% TBSA of deep burns in children, they are considered as severe burn and can induce multiple organ injury and excessive systemic inflammation. Severe burn is a complex and severe traumatic injury with high mortality in the acute phase, and prompt and appropriate treatment of severe burns is necessary, especially in the phase.

Mesenchymal stem cells (MSCs) are considered as perfect candidate for cell-based therapy and regenerative medicine. And as an excellent representative of MSCs, the human umbilical cord MSCs (hUCMSCs) have a lot of advantages, such as short amplification time, high proliferation rate, lower immunogenicity, higher safety, abundance, and convenience compared with other original MSCs. In our previous studies, we found that hUCMSCs transplantation could accelerate severely burned wound healing and alleviate severe burn-induced acute lung injury in rats [20, 27]. Choi et al.'s study also showed that combination therapies of MSCs and low-flow extracorporeal life support (ECLS) were very effective on acute respiratory distress syndrome (ARDS) and MOF due to inhalation injury and burns [9]. Furthermore, increasing evidence has shown that hUCMSCs can protect the severe burn and traumatic brain injury (TBI) induced multiple organ dysfunction mainly through secretion of bioactive factors, such as antiapoptotic factors, immunomodulation factors, antioxidant factors, and exosomes [28–30]. In this study, we found that hUCMSCs administration preserved function of vital organs, such as the heart, kidney, and liver, and ameliorated histopathological alterations induced by severe burn. The possible mechanisms underlying the effect of hUCMSCs administration involved the encouraging hUCMSCs migration, increasing level of protective cytokine IGF-1 and ratio of BCL-2/BAX, and then decreasing cell apoptosis rates in these major organs.

Firstly, we obtained hUCMSCs still using the tissue block attachment method according to the previous procedure [31]. Similarly, the cells were identified by flow cytometer and labeled with green fluorescent protein (GFP) using a lentiviral strategy. The identification results of flow cytometer were consistent with the immune-phenotypic markers of MSCs. Moreover, after GFP-labeled hUCMSCs transplantation, we further characterized the effects of the hUCMSCs administration on organ structures and functions in early stage post severe burn, along with the potential mechanisms associated with the therapeutic effects.

Next, we found that administrated hUCMSCs largely migrated into the blood vessel of injured organs with blood flow at 24 hours post severe burn, and a very few cells were present in these organ tissues. The MSCs migration into these damaged organs, which were likely involved in the complex process regarding their trafficking, extravasation, homing, and migration after the cell transplantation, and some chemokines, such as SDF-1, CCR1, CCL25, and CX3CL1, might play a key recruitment role in critical phases [32–34].

Our results also showed that hUCMSCs administration significantly improved severe burn-induced structure damages and reduced cell apoptosis rates in the heart, kidney, and liver. Furthermore, the biochemistry parameters of myocardial damage, liver function, and kidney function in serum were explicitly increased post severe burn, and they also were significantly decreased by hUCMSCs administration. Therefore, the hUCMSCs could better protect the organ structures and functions at early stage post severe burn. And other preclinical studies also have shown hMSCs therapeutic value in studies of myocardial infarction, diabetes, sepsis, hepatic failure, acute renal failure, and acute lung injury (ALI) [35–39]. But administered hUCMSCs do not massively integrate into the injured tissues, so we speculated reasonably the main mechanism by which hUCMSCs play a role in repairing damaged tissues by paracrine protective cytokines. It is well recognized that IGF-1 is an important protective factor and BCL-2 is a key antiapoptosis protein, which can inhibit the apoptotic progress and accelerate injured organ regeneration [40, 41]. Therefore, we detected levels of the representative and protective cytokines, such as IGF-1, BCL-2, and BAX in these vital organs, including the heart, kidney, and liver [41]. Our results also showed that hUCMSCs administration significantly increased IGF-1 level and upregulated ratio of BCL-2 and BAX compared to the burn group.

In conclusion, hUCMSCs transplantation attenuated multiple organ injuries and preserved these organ functions in early stage post severe burn by encouraging the migration of hUCMSCs, increasing level of IGF-1 protective cytokine and ratio of BCL-2/BAX, and subsequently decreasing cell apoptosis and injuries of these major organs in severely burned rats.

## Figures and Tables

**Table 1:** The hUCMSC administrations significantly improved parameters of the heart, liver, and kidney functions. Serological biochemical analysis showed that high sensitive parameters of myocardial damage, such as troponin, CK, and CK-MB levels in the burn groups, were explicitly increased than those in the sham groups, and they were significantly decreased by hUCMSC administrations. The levels of liver function parameters ALT, AST, and LDH and kidney function parameters urea and creatinine in the burn groups were markedly higher than those in the sham groups, and their levels in the burn+hUCMSC groups were significantly lower than those in the burn groups.

	Sham	Burn	Burn+hUCMSCs
Troponin (pg/mL)	216.23 ± 12.47	304.35 ± 14.21^@@^	250.77 ± 4.03^∗∗^
CK (U/L)	1235.45 ± 286.50	5681.75 ± 264.05^@@^	3011.33 ± 338.83^∗∗^
CK-MB (U/L)	1418.67 ± 271.34	3421.75 ± 249.87^@@^	2028.25 ± 62.24^∗∗^
LDH (U/L)	1309.12 ± 263.24	3176.88 ± 175.37^@@^	1932.04 ± 59.48^∗∗^
ALT (U/L)	41.34 ± 4.89	199.11 ± 32.41^@@^	106.33 ± 6.66^∗∗^
AST (U/L)	126.00 ± 37.66	832.78 ± 30.49^@@^	497.00 ± 10.41^∗∗^
Urea (mmol/L)	4.24 ± 0.60	12.44 ± 0.43^@@^	7.91 ± 0.82^∗∗^
Creatinine (*μ*mol/L)	17.08 ± 0.81	28.07 ± 0.44^@@^	21.67 ± 0.26^∗∗^

^@^ *P* < 0.05 and ^@@^*P* < 0.01 compared with the sham group, respectively. ^∗^*P* < 0.05 and ^∗∗^*P* < 0.01 compared with the burn group, respectively.

## Data Availability

All raw data for this article can be obtained by contacting the corresponding author, Dr. Lingying Liu.

## Additional Points

*Statements.* (1) All of the studies covered in this article followed compliance with the Helsinki Congress and the Declaration of Istanbul. (2) The use of prisoners was not involved in the study, and none of the participants received payment or coercion.
